# Self-Interested Framed and Prosocially Framed Messaging Can Equally Promote COVID-19 Prevention Intention: A Replication and Extension of Jordan et al.’s Study (2020) in the Japanese Context

**DOI:** 10.3389/fpsyg.2021.605059

**Published:** 2021-05-11

**Authors:** Takeru Miyajima, Fumio Murakami

**Affiliations:** Faculty of Social Studies, Nara University, Nara, Japan

**Keywords:** COVID-19, persuasion, messaging, self-interest, collective-interest, culture

## Abstract

How can we effectively promote the public’s prevention of coronavirus disease 2019 (COVID-19) infection? Jordan et al. (2020) found with United States samples that emphasizing either self-interest or collective-interest of prevention behaviors could promote the public’s prevention intention. Moreover, prosocially framed messaging was more effective in motivating prevention intention than self-interested messaging. A dual consideration of both cultural psychology and the literature on *personalized matching* suggests the findings of Jordan et al. (2020) are counterintuitive, because persuasion is most effective when the frame of the message delivered and the recipient of the message are culturally congruent. In order to better understand the potential influence of culture, the current research aimed to replicate and extend Jordan et al. (2020) findings in the Japanese context. Specifically, we examined the question (1) whether the relative effectiveness of the prosocial appeal is culturally universal and robust, (2) which types of *‘others’* especially promote prevention intention, and (3) which psychological mechanisms can explain the impact of messaging on prevention intention. In Study 1 (*N* = 1,583), we confirmed that self-interested framed, prosocially framed, and the combination of both types of messaging were equally effective in motivating prevention intention. In Study 2 (*N* = 1,686), we found that family-framed messaging also had a promoting effect similar to that from self-interested and prosocial appeals. However, the relative advantage of prosocial appeals was not observed. Further, a psychological propensity relevant to sensitivity to social rejection did not moderate the impact of messaging on prevention intention in both studies. These results suggest that since engaging in the infection control itself was regarded as critical by citizens after public awareness of COVID-19 prevention has been sufficiently heightened, for *whom* we should act might not have mattered. Further, concerns for social rejection might have had less impact on the prevention intentions under these circumstances. These results suggest that the relative advantage of a prosocial appeal might not be either culturally universal or prominent in a collectivistic culture. Instead, they suggest that the advantages of such an appeal depends on the more dynamic influence of COVID-19 infection.

## Introduction

The coronavirus disease 2019 (COVID-19) pandemic poses an enormous threat to our lives. As it is highly contagious, successfully motivating the public to actively engage in preventing infection is key to slowing down interpersonal transmission. To manage the pandemic and its impact, it is imperative to elucidate an effective intervention strategy that promotes individual infection prevention behaviors supported by behavioral and social sciences (Van Bavel et al., 2020).

Accordingly, some scholars have suggested that not only self-interested framed messaging (i.e., highlighting the threat to themselves and encouraging prevention behaviors), but also collective-interested framed messaging (i.e., highlighting the threat to others or the community and encouraging prevention behaviors) can motivate the public’s prevention behaviors (Capraro and Barcelo, 2020; Jordan et al., 2020; Pink et al., 2020; Sasaki et al., 2020; Heffner et al., 2021). Some studies have directly compared the effectiveness of those messages on prevention intentions, suggesting the relative advantage of prosocially framed messaging (Capraro and Barcelo, 2020; Jordan et al., 2020; Sasaki et al., 2020). Specifically, Jordan et al. (2020) conducted experiments in the early stage of the COVID-19 pandemic (i.e., March 14–16, 2020) and the later stage (i.e., April 17–30) with United States samples. They found that participants assigned to either public (i.e., exposed to the message emphasizing the public benefit of prevention) or personal + public condition (i.e., exposed to the message emphasizing both personal and public benefits of prevention) showed greater intention to engage in prevention behaviors than those assigned only to the personal condition (i.e., exposed to the message emphasizing the personal benefit of prevention) in the earlier set of studies. However, no differences in the effectiveness of self-interested versus prosocial appeals were observed in the later set of studies. Despite the inconsistent results on the relative advantage of prosocial appeals, exposure to the message was more effective in increasing prevention intention than baselines.

Although these findings provide great insights into how to confront COVID-19, they simultaneously raise some questions: whether the relative effectiveness of the prosocial appeal is culturally universal and robust, which types of “*others*” especially promote prevention intention, and which psychological mechanisms can explain the impact of messaging on prevention intention.

Regarding the first question, most of these studies were examined in Western, educated, industrialized, rich, and democratic samples (except Sasaki et al., 2020). Results obtained from an extremely narrow cultural population do not necessarily ensure similar results in a broader population. Cross-cultural studies find that people in individualistic/collectivistic cultures show different psychological processes, such as self-construal, the nature of relationships with others, and cognitive style (e.g., Triandis, 1995; Oyserman et al., 2002). In individualistic cultures, the core element is the individual. Individuals are independent of one another and detached from their collectives. Conversely, in collectivistic cultures, the core element is the group. Individuals are seen as fundamentally bound in groups and associated through their group memberships. This cultural dimension is considered influential in exploring the cross-cultural differences across various countries and regions. Thus, researchers should continue to investigate the cross-cultural universality and robustness of findings across different cultural contexts.

Previous literature in the domains of marketing and health communication demonstrates that persuasion is most effective when the frame of the message delivered and the recipient of the message are culturally congruent (see Rodrigues et al., 2018; Teeny et al., 2020, for a review). For instance, Uskul and Oyserman (2010) find that European Americans who were primed for individualism were more likely to accept the message when it focused on individual physical consequences. In a similar vein, the message was more persuasive when Asian Americans who were primed for collectivism received a message focused on relational obligation. Spina et al. (2018) demonstrates that, when Latina women were exposed to a family-focused message, collectivistic and familial values positively predicted intentions to undergo cervical cancer screening, whereas these values did not predict intentions among those who were exposed to the self-focused message. Considering the literature on *personalized matching*, the findings of Jordan et al. (2020) may be counterintuitive. That is, we can predict that the self-interested framed messaging would be more effective than collective-interested framed messaging among American samples, among which individualism is relatively prevalent. However, the effect of collective interest–framed messaging would be greater than self-interest–framed messaging among Japanese samples, among which collectivism is relatively dominant. One study that investigated the impact of self-interested framed messages and prosocially framed messages on COVID-19 prevention with a Japanese sample showed mixed results (Sasaki et al., 2020). In their study, although altruistic messaging (i.e., emphasizing the threat to close others and encouraging prevention behaviors) partially amplified prevention intentions, the self-reported behavioral changes for prevention were not actually observed when measured after the experimental intervention. In contrast, most of the messages (i.e., altruistic, self-focused, and altruistic + self-focused) decreased the frequency of some of the prevention behaviors. Therefore, we cannot conclude whether prosocially framed messages are more effective than self-focused ones universally or the relative advantage of specific framed messaging would vary across cultures.

With respect to the second question, although there are multiple types of interpersonal relationships, those that could lead to prevention behaviors more effectively remain unclear. Considering the practical significance of the messaging, it is beneficial to examine whether different types of “*others*” affect the effectiveness of the message. Indeed, some scholars argue that the effect of family-framed messaging should be explored in future research (Everett et al., 2020; Jordan et al., 2020). However, it does not answer how effective the message may be if it specifically focuses on the benefits to one’s own family. It is not surprising that emphasizing the benefits to one’s loved ones may motivate one to act for their sake, an idea also endorsed by evolutionary psychology (e.g., Krupp et al., 2008). Korchmaros and Kenny (2001) demonstrate that individuals were more willing to act altruistically toward others with whom they shared a higher degree of genetic relatedness. Madsen et al. (2007) support this notion by experimentally assessing the impact of kinship on altruistic behavior. They suggest that people act more altruistically toward more biologically related individuals. These arguments suggest that family-framed messaging would motivate people’s prevention intentions more strongly than any other condition.

Regarding the third question, little is known about the psychological mechanism underlying the relative effectiveness of prosocial appeals. One potential mechanism may be relevant to the prosocial emotional process: empathy for other people. People often act for the welfare of others regardless of their closeness in terms of their relationships. Caring for both self- and collective interest is supposed to be a fundamental human motive (e.g., Fehr and Fischbacher, 2003). This notion is supported by some empirical literature in which empathy is related to COVID-19 prevention behaviors (Christner et al., in press; Lunn et al., 2020; Pfattheicher et al., 2020). Thus, exposure to social relation cues may have activated empathy toward others, leading individuals to engage in collective-interested behavior (i.e., prevention behavior) during the COVID-19 pandemic. Here, we consider that messages highlighting the importance of prevention behavior to protect others might potentially deliver another cue that is relevant to acting as a responsible citizen for the community and sanctions against deviance. That is, the prosocial message may act as a cue to make the individual aware of adherence to social norms.

Social scientists repeatedly demonstrate that social norms often dictate individual judgments and behaviors (e.g., Cialdini and Goldstein, 2004). The strength of social norms varies across cultures. More specifically, nations in East Asia have strict social norms and punishments for norm violations, and those in North America have weaker norms and are more tolerant of deviance (Gelfand et al., 2011, 2017). Supporting this notion, Nakayachi et al. (2020) reveal that perceived social norms were associated with the frequency of mask wearing more strongly than the motivation to reduce the risk of infection for the self and others in the Japanese sample during the COVID-19 pandemic. In contrast, Bilancini et al. (2020) show, with a sample from Italy, where individualism is relatively more prevalent and norm-deviance is more permissive (Gelfand et al., 2011), that there was no significant effect of making a specific type of norm (e.g., descriptive norm, injunctive norm) more salient to ensure careful attention and comprehension of the information about behaviors recommended by the administration. Thus, the effectiveness of prosocially framed messaging might be attributed not only to the activation of prosocial motives as a moral actor, but also the motive of avoiding social rejection due to a lack of adherence to normative behaviors in the immediate community.

If prosocial appeals induce one’s sense of compliance to perceived social norms, the impact of the other-focused message on prevention behavior should be more pronounced among individuals who are more susceptible to social rejection. Previous literature suggests that there is a cultural variation in rejection sensitivity. Specifically, in line with the aforementioned arguments by Gelfand et al. (2011), East Asians show a greater extent of interpersonal rejection sensitivity than North Americans (e.g., Yamaguchi et al., 1995). Yuki and Schug (2020) explain the cultural difference in this psychological tendency via a social-ecological factor: relational mobility. Relational mobility is defined as the number of opportunities people have in a given society or social context to select new relationship partners when necessary (Yuki et al., 2007). Some studies confirm that individuals in a low relational mobility society are likely to be more sensitive to interpersonal rejection (Sato et al., 2014; Lou and Li, 2017). In societies with low relational mobility, as individuals are embedded in relatively fixed social networks, they are driven to monitor social cues and social norms so that they can behave appropriately and minimize the possibility of being rejected from the current social relationship (Yuki and Schug, 2020). Drawing on the cultural variation of rejection sensitivity, if this psychological propensity is combined with the relative advantage of prosocial appeals, it should be prominent, especially among East Asians. Thus, attempts to investigate this hypothesis could be crucial to unpack the cultural mechanism of the relative effectiveness of prosocially framed messaging on prevention behaviors.

## The Present Research

The current research has three primary purposes. First, we examined the cultural universality and robustness of the findings of Jordan et al. (2020), which verified the relative advancement of prosocially framed over self-interested framed messages in non-White samples. Second, we sought to extend their findings by examining another type of “*other*”-focused message. Third, we aimed to reveal one of the psychological mechanisms underlying the relative effectiveness of prosocial appeals.

Specifically, Study 1 was designed to directly replicate previous studies conducted in the United States. In Study 2, we tested the effect of family-framed messaging (i.e., emphasizing the COVID-19 threat to the family) on prevention intentions. We further explored whether the messaging effects were moderated by participants’ perceptions of relational mobility. Moreover, we also measured an individual’s fear of negative evaluation from others (FNE) as an individual difference in rejection sensitivity so that we could explore its moderating effect more directly. Yuki and Schug (2020) indicate that the extent of perceived relational mobility differs not only between major regions (e.g., North America vs. East Asia) but also within the same country (e.g., urban vs. rural). Here, the comparison between the North American and East Asian samples appears to be convenient for hypothesis verification. However, the current COVID-19 situation differs greatly across countries. Given this situation, testing the moderating role of relational mobility on the relationship between messaging and prevention intention by comparing samples from two different countries may involve the challenge of ruling out potential confounding factors. Hence, we sought to test the hypothesis only with Japanese citizens living in Japan because the current situation of infection did not differ significantly.

Furthermore, to eliminate the alternative explanation of the previous findings that prosocially framed messages merely induced socially desirable responses, we measured the social desirability score and attempted to control the potential confounding effect of social desirability bias. The experimental material, items, and raw data are available through the Open Science Framework^1^. All analyses were performed using HAD 16.302 (Shimizu, 2016).

## Study 1

In Study 1, we attempted to replicate the Jordan et al. (2020) findings in the later stage (i.e., May 22–23, 2020) of the first wave of the pandemic in the Japanese context. The situation in Japan at that time was as follows: The number of confirmed cases was more than 16,000, and deaths were fewer than 800. The nationwide declaration of a state of emergency was lifted except in a few prefectures (i.e., Hokkaido, Saitama, Chiba, Tokyo, and Kanagawa).

### Method

#### Participants

We recruited Japanese citizens living in Japan aged over 18 years via a Japanese crowdsourcing service, CrowdWorks^2^ from May 22 to May 23, 2020. We obtained 1,627 participants in exchange for 100 JPY (roughly US$0.93). Forty-two participants failed an attention check question (ACQ; Oppenheimer et al., 2009; “Please select option 7 for this item”), and two participants did not identify themselves as Japanese. After excluding these participants, we included 1,583 participants in the final analysis (male = 574, female = 1,009, *M*_*age*_ = 37.90, *SD* = 10.01).

Of the sample, 49.34% were married, the average number of children was 0.63 (*SD* = 0.97), and 67.78% were currently employed (40.18% were *others*, 9.92% were *service industries*, and 7.58% were *manufacturing*). Responses were obtained from citizens of all 47 prefectures although the percentage of participants from prefectures with large populations was relatively high (Tokyo = 15.67%, Kanagawa = 9.10%, Osaka = 7.71%).

To test the effect of message framing (i.e., personal vs. public vs. personal + public vs. control) on the prevention intention and perceived threat of COVID-19, a one-way ANOVA was employed. A power analysis using G^∗^Power 3.1 (Faul et al., 2007) showed that the required sample size was 1,096 to detect a small main effect (i.e., *f* = 0.10) with α = 0.05 and power (1 − β) = 0.80. We also tested the hypothesized interaction between condition and either relational mobility or FNE on prevention intention. A power analysis showed that 1,095 was required to detect a small interaction effect (i.e., *f* = 0.10) in either a 4 (condition: personal vs. public vs. personal + public vs. control) × 2 (relational mobility: high vs. low) between-factorial design ANOVA or a 4 (condition: personal vs. public vs. personal + public vs. control) × 2 (FNE: high vs. low) between-factorial design ANOVA with α = 0.05 and power (1 − β) = 0.80.

#### Procedures

Similar to the Jordan et al. (2020) study, participants were randomly assigned to one of four experimental conditions, which consisted of a control condition (involving no treatment) and the three treatment conditions (personal, public, and personal + public). Consent from all participants was obtained prior to the experiment, after which we began by exposing participants in the treatment conditions to the relevant treatment. Participants in the control condition advanced to the items to measure prevention intentions immediately after the consent form.

**FIGURE 1:**
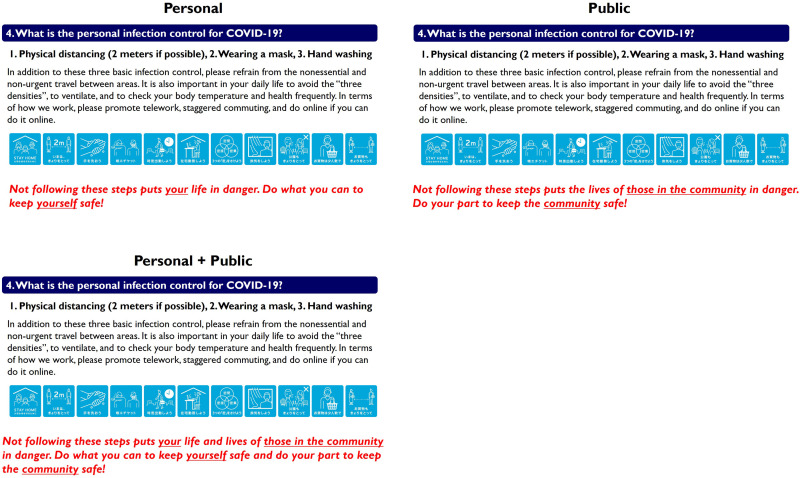
Messages shown in each treatment.

In all three treatments, participants were presented with three slides with illustrations and text explanations in sequence. Drawing on public information from the Line News (2020), Ministry of Health, Labour and Welfare (2020), and the Prime Minister’s Official Residence (2020), the authors created slides to explain the current situation regarding COVID-19^3^. The slides briefly explained basic information about COVID-19, and participants were asked to read them carefully. The illustrations and text explanations in the slides were identical across treatments; only the message aimed at participants and shown in the third slide varied across treatments (Figure 1). To ensure that the message content was delivered to participants, the message was written in red and bold, and the subject of the action (i.e., personal or community) was underlined. The messages for each treatment were as follows:

*Personal: Not following these steps puts your life in danger. Do what you can to keep yourself safe!*

*Public: Not following these steps puts the lives of those in the community in danger. Do your part to keep the community safe!*

*Personal* + *Public: Not following these steps puts your life and lives of those in the community in danger. Do what you can to keep yourself safe and do your part to keep the community safe!*

After the participants were exposed to the slides, we presented four questions about the content of the slides and indicated that they select the correct option for each question. These were prepared to confirm whether the participants had concentrated on and accurately understood the information earlier. If the participants failed to complete any one of these questions, they were supposed to be exposed to the slides again. That is, the participants who failed to answer any question were obliged to read the slides until they completed all four questions. In the fourth question, participants were asked to answer about the correct message displayed on the last slide (See supplementary information in OSF for details). Therefore, answering this question correctly indicated that participants could understand and remember the experimental treatment accurately. In addition to the ACQ, these procedures were adopted to eliminate *satisficing* (Krosnick, 1991), which refers to behaviors by which participants complete survey questions without sufficient cognitive effort. Some studies have documented that satisficing could deteriorate the quality of data and distort the results (Miura and Kobayashi, 2016, 2019). Thus, we included items and procedures designed to minimize satisficing.

#### Measures

##### Prevention intentions

Participants reported their intentions to engage in a series of 15 prevention behaviors (e.g., “*To avoid going to places with poor ventilation*”) on a 0–100 scale (0 = strongly disagree, 50 = neither agree nor disagree, 100 = strongly agree). These items consisted of several categories on infection prevention (e.g., personal hygiene, interpersonal contact, social distancing, and self-isolation). The items were created by the authors by referring to the items used in a national survey conducted by the Japanese Ministry of Health, Labor and Welfare and the LINE corporation, and behavior patterns introduced as the “new lifestyle” by an expert meeting on COVID-19 in Japan. To create a prevention intention score (α = 0.930), we calculated the average of the 15 items. Higher scores indicated that participants intended to engage in more prevention behaviors hereafter.

##### Perceived threat of coronavirus

We measured the perceived personal threat (i.e., a threat to the participant; α = 0.880), family threat (to the participant’s family; α = 0.937), and public threat (to the community; α = 0.927) with two items for each type of threat created by the authors. These items were presented to participants in a fixed order. Participants indicated their perceived threat to themselves (e.g., “*Considering the impact on yourself, to what extent are you afraid of contracting the new coronavirus?*”), threat to their family (e.g., “*Considering the impact on your family, to what extent are you afraid of contracting or spreading the new coronavirus?*”), and threat to the community (e.g., “*Considering the impact on your community, to what extent are you afraid of contracting or spreading the new coronavirus?*”) on a 0–100 scale (e.g., 0 = not at all, 50 = to a moderate extent, 100 = to an enormous extent). Higher scores indicate that the participants perceived a greater threat of coronavirus for each target.

##### Relational mobility

Participants indicated their perceptions of the relational mobility of their immediate society (e.g., “*They have many chances to get to know other people.*”) on a 6-point scale (1 = strongly disagree to 6 = strongly agree) with the Relational Mobility Scale (Yuki et al., 2007). We calculated the average of the 12 items to create a relational mobility score (α = 0.835). Higher scores indicated that participants perceived more flexibility in the nature of interpersonal relationships in their immediate society.

##### Fear of negative evaluation from others

Participants reported their social-evaluative anxiety (e.g., “*Even though I know that it doesn’t matter what people think, I worry about what people think about me*”) on a 5-point scale (1 = strongly disagree to 5 = strongly agree) with the short version of the Fear of Negative Evaluation Scale for Japanese (Sasagawa et al., 2004). We calculated the average of the 12 items to create an FNE score (α = 0.949). Higher scores indicated that participants showed greater anxiety about being evaluated negatively by others.

##### Social desirability

Participants completed the Japanese version of the Balanced Inventory of Desirable Responding (e.g., “*I don’t regret the decisions I’ve made.*”; BIDR-J; Tani, 2008) on a 7-point scale (1 = strongly disagree to 7 = strongly agree). Here, this scale does not mean participants’ tendency to view the issues at hand as socially desirable, but rather a personality tendency to respond in more socially desirable ways generally. To create a composite measure of social desirability, we averaged the responses for 24 items (α = 0.805). Higher scores reflected higher levels of social desirability.

##### Demographic variables

We recorded participants’ demographic information. Specifically, participants reported their age, gender, marital status, number of children, current employment status, occupation, and residential area. In addition, their personal health conditions and health conditions of the family were recorded (see supplementary information in OSF for details).

### Results

Prior to analyzing the effect of messaging, we checked whether several demographic characteristics relevant to dependent variables differed between conditions. A one-way ANOVA revealed that there were no significant differences in the mean age between conditions, *F*(3,1579) = 0.99, *p* = 0.395, $\eta_{\text{p}}^{2}$ = 0.002, 95% CI [0.000, 0.007]. Neither gender ratio (χ^2^[3, *N* = 1583] = 3.89, *p* = 0.273) nor residential area (χ^2^[138, *N* = 1583] = 118.81, *p* = 0.880) showed significant bias between conditions. In addition, a significant imbalance between conditions on participants’ personal health condition (i.e., number of chronic diseases associated with COVID-19 aggravation), *F*(3,1579) = 0.54, *p* = 0.654, $\eta_{\text{p}}^{2}$ = 0.001, 95% CI [0.000, 0.004], and the health conditions of their family, χ^2^(3, *N* = 1583) = 1.59, *p* = 0.661, were not confirmed.

**FIGURE 2:**
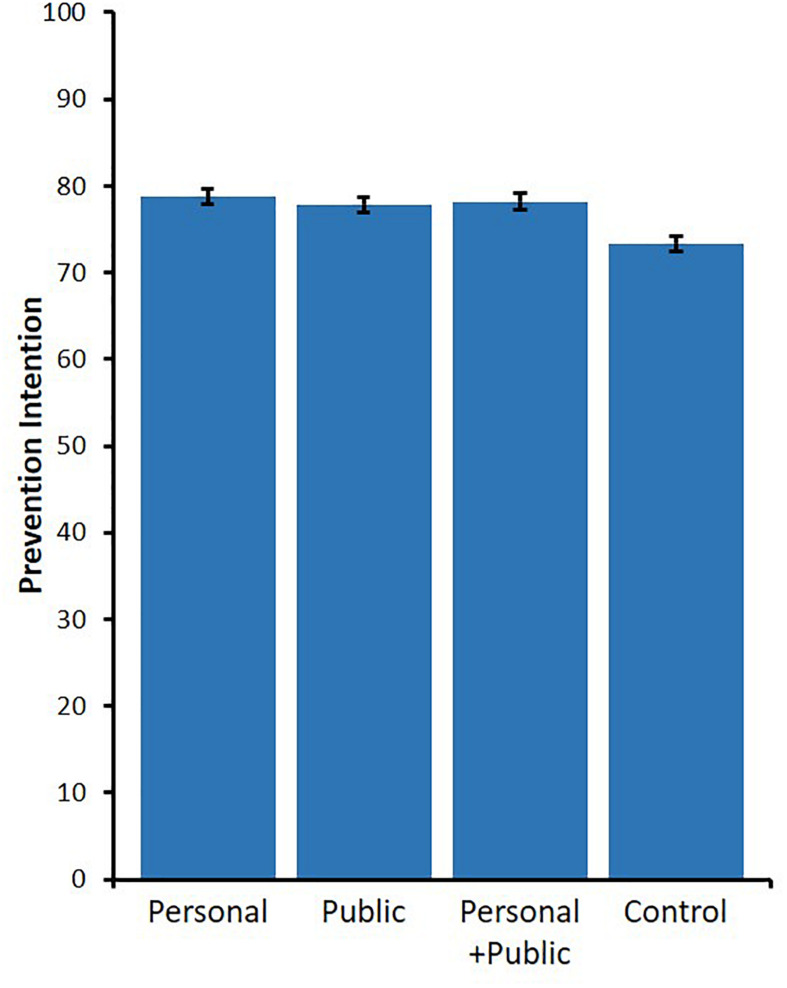
Means of prevention intention per treatments in Study 1 (error bars depict standard error).

We performed a one-way ANOVA to test the effect of message framing on prevention intention. The main effect of message framing was significant, *F*(3,1579) = 8.14, *p* < 0.001, $\eta_{\text{p}}^{2}$ = 0.015, 95% CI [0.005, 0.028] (Figure 2). Multiple comparisons using Holm’s method demonstrate that the mean levels of all three treatment conditions were significantly higher than those of the control condition (*M* = 73.36, *SD* = 18.02), vs. personal condition: *M* = 78.78, *SD* = 17.09, *t*[1579] = 4.44, *p* < 0.001, *d* = 0.308, 95% CI [0.169, 0.446]; vs. public condition: *M* = 77.84, *SD* = 16.95, *t*[1579] = 3.61, *p* = 0.001, *d* = 0.254, 95% CI [0.116, 0.392]; vs. personal + public condition: *M* = 78.19, *SD* = 18.43, *t*[1579] = 3.79, *p* < 0.001, *d* = 0.274, 95% CI [0.136, 0.412]. Contrary to our prediction, there were no significant differences among the three treatments, *p*s > 0.784, *d*s < 0.053.

Furthermore, we checked whether social desirability would alter these results. Specifically, we conducted a one-way analysis of covariance (ANCOVA), which included social desirability in the model as a covariate. Significant main effects of both condition, *F*(3,1578) = 6.99, *p* < 0.001, $\eta_{\text{p}}^{2}$ = 0.013, 95% CI [0.003, 0.025] and social desirability, *F*(1,1578) = 30.66, *p* < 0.001, $\eta_{\text{p}}^{2}$ = 0.019, 95% CI [0.008, 0.034] were observed. Next, multiple comparisons were conducted using Holm’s method. In accordance with the result of ANOVA, the mean levels of all three treatment conditions were significantly higher than those in the control condition (*M* = 73.66, *SD* = 17.48) vs. personal condition: *M* = 78.73, *SD* = 17.45, *t*[1578] = 4.18, *p* < 0.001, *d* = 0.290, 95% CI [0.152, 0.428]; vs. public condition: *M* = 77.79, *SD* = 17.45, *t*[1578] = 3.34, *p* = 0.003, *d* = 0.236, 95% CI [0.098, 0.374]; vs. personal + public condition: *M* = 77.97, *SD* = 17.47, *t*[1578] = 3.40, *p* = 0.003, *d* = 0.246, 95% CI [0.109, 0.384]. Again, there were no significant differences between the three treatments, *p*s > 0.886, *d*s < 0.054.

Next, to investigate whether exposure to the message increased the perceived threat of COVID-19, we compared the means of each condition by separate one-way ANOVAs for each of the three types of threat (i.e., personal, family, and public). The main effect of messaging was not significant for any of the three: personal, *F*(3,1579) = 1.54, *p* = 0.201, $\eta_{\text{p}}^{2}$ = 0.003, 95% CI [0.000, 0.009]; family, *F*(3,1579) = 0.62, *p* = 0.600, $\eta_{\text{p}}^{2}$ = 0.001, 95% CI [0.000, 0.005]; or public, *F*(3,1579) = 2.51, *p* = 0.057, $\eta_{\text{p}}^{2}$ = 0.005, 95% CI [0.000, 0.012].

Finally, we sought to explore the moderating role of relational mobility and FNE on the relationship between message framing and prevention intentions. To investigate the moderating roles, dummy-coded relational mobility scores and dummy-coded FNE scores were created by splitting the variables into two groups by median (1 = high, 0 = low). First, a two-way ANOVA (4 [condition: personal vs. public vs. personal + public vs. control] × 2 [relational mobility: high vs. low]) was performed. The results showed a significant main effect for the condition, *F*(3,1575) = 8.15, *p* < 0.001, $\eta_{\text{p}}^{2}$ = 0.015, 95% CI [0.005, 0.028], but no significant main effect for dummy-coded relational mobility, *F*(1,1575) = 1.04, *p* = 0.308, $\eta_{\text{p}}^{2}$ = 0.001, 95% CI [0.000, 0.006], nor a significant interaction, *F*(3,1575) = 1.41, *p* = 0.239, $\eta_{\text{p}}^{2}$ = 0.003, 95% CI [0.000, 0.008]. In the same manner, a two-way ANOVA (4 [condition: personal vs. public vs. personal + public vs. control] × 2 [FNE: high vs. low]) on prevention intention was performed. The results showed a significant main effect for the condition, *F*(3,1575) = 8.69, *p* < 0.001, $\eta_{\text{p}}^{2}$ = 0.016, 95% CI [0.005, 0.029], but no significant main effect for dummy-coded FNE, *F*(1,1575) = 0.51, *p* = 0.474, $\eta_{\text{p}}^{2}$ = 0.000, 95% CI [0.000, 0.004], and no significant interaction, *F*(3,1575) = 2.41, *p* = 0.065, $\eta_{\text{p}}^{2}$ = 0.005, 95% CI [0.000, 0.012]. In sum, the effect of messaging on prevention intention was not moderated by these variables^4^.

## Discussion

Study 1 sought to provide a conceptual replication of Jordan et al. (2020) in the Japanese context. Specifically, we examined whether presenting prevention messages enhances the public’s prevention intentions. Moreover, we investigated the hypothesis that this effect was strengthened when people were exposed to other-oriented framing messages rather than self-oriented ones. We found that exposing people to prevention messages promotes their prevention intentions more effectively compared with not exposing them to messages; however, the relative advantage of prosocial appeals was not obtained. Although our results were not consistent with the earlier set of studies by Jordan et al. (2020), their later set of studies demonstrated the same pattern as this study.

The experimental treatments did not amplify the perceived threat of the coronavirus. These results are also in line with the findings of Jordan et al. (2020). These findings strongly support the idea that delivering messages increases prevention intentions, not because they escalate the perceived threat of the coronavirus. In addition, social desirability could not explain the differences between the conditions. This suggests that the message effect does not reflect mere activation of participants’ bias to appear socially desirable.

Neither relational mobility nor FNE played a moderating role in the treatment–prevention relationship. Given the results of this study, a normative explanation for the effectiveness of prosocial framing might not be strongly supported.

## Study 2

Study 2 was designed to replicate Study 1 and extend the findings of Jordan et al. (2020). Specifically, we included the “family” condition instead of the “personal + public” condition. This study was conducted in the later stage (i.e., May 28–30, 2020) of the first wave of the pandemic in the Japanese context. The situation in Japan at that time was as follows: the number of confirmed cases was more than 16,500, and deaths exceeded 800. The declaration of a state of emergency was lifted nationwide on May 25.

### Method

#### Participants

Japanese citizens living in Japan aged over 18 years were recruited via a Japanese crowdsourcing service, CrowdWorks, from May 28 to 30, 2020. We obtained 1,746 participants in exchange for 100 JPY (roughly US$0.93). Fifty-six participants failed an ACQ, and three participants did not identify themselves as Japanese. After excluding these data^5^, we included 1,686 participants in the final analysis (male = 546, female = 1,140, *M*_*age*_ = 36.27, *SD* = 10.46).

Of the sample, 49.47% were married, the average number of children was 0.66 (*SD* = 1.05), and 69.57% were currently employed (37.84% were *others*, 10.44% were *service industries*, 8.24% were *manufacturing*). Here again, responses were obtained from the citizens of all 47 prefectures. The percentage of participants from prefectures with large populations was still relatively high (Tokyo = 15.72%, Osaka = 8.54%, Kanagawa = 8.07%).

The sample size was determined before data collection using a power analysis with G^∗^Power 3.1 (Faul et al., 2007), which required the same sizes because of the identical factorial designs as in Study 1. Note that participants in Study 1 were not allowed to participate in Study 2 systematically; as such, there was no duplication of participants in the studies.

#### Procedures

**FIGURE 3:**
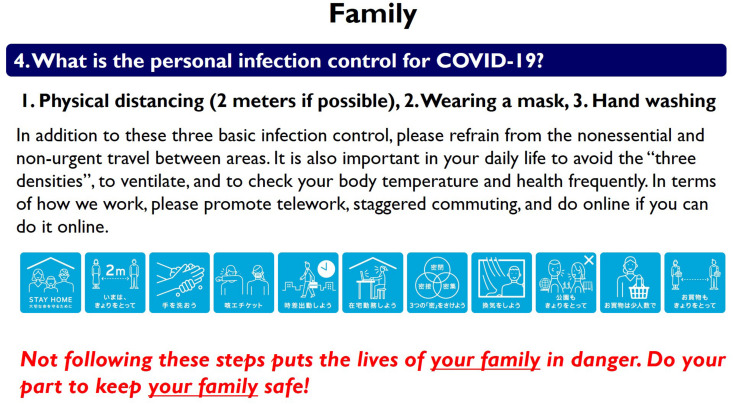
Message shown in *Family* treatment.

The experimental procedure was almost the same as that in Study 1. There were again four conditions, which consisted of a control condition (involving no treatment) and the three treatment conditions (personal, public, and family). The messaging slides for family treatment are shown in Figure 3; the message was as follows:

*Family: Not following these steps puts the lives of your family in danger. Do your part to keep your family safe!*

Considering the change in the number of cases and the current situation in Japan, the information depicted in the slides was partially modified (see supplementary information in OSF for detailed information).

#### Measures

The same scales and items from Study 1 were used in Study 2. Specifically, we measured prevention intention (α = 0.923), perceived personal (α = 0.863), family (α = 0.927), public threat of the coronavirus (α = 0.921), relational mobility (α = 0.824), FNE (α = 0.945), social desirability (α = 0.793), and demographic variables. All variables except for demographic variables were averaged in the same manner as in Study 1 and used in the subsequent analysis.

### Results

Prior to the analysis to test the hypothesis, we examined the imbalance in several demographic characteristics between conditions. One-way ANOVA showed that there were no significant differences in the mean age between conditions, *F*(3,1682) = 0.36, *p* = 0.783, $\eta_{\text{p}}^{2}$ = 0.001, 95% CI [0.000, 0.003]. Neither the gender ratio (χ^2^[3, *N* = 1686] = 0.98, *p* = 0.806) nor residential area (χ^2^[138, *N* = 1686] = 131.71, *p* = 0.635) demonstrated significant bias between conditions. Furthermore, the significant imbalance between conditions on participants’ personal health conditions, *F*(3,1682) = 0.93, *p* = 0.424, $\eta_{\text{p}}^{2}$ = 0.002, 95% CI [0.000, 0.006], and the health conditions of their family, χ^2^(3, *N* = 1686) = 3.26, *p* = 0.353, were not confirmed.

**FIGURE 4:**
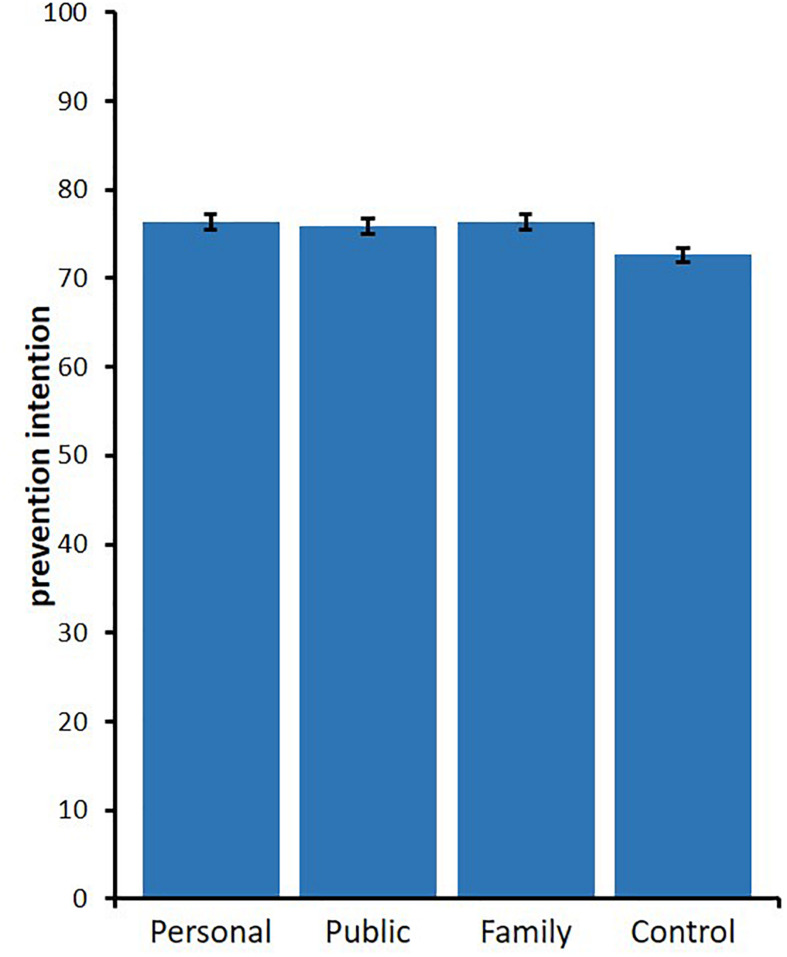
Means of prevention intention per treatments in Study 2 (error bars depict standard error).

A one-way ANOVA was employed to test the effect of message framing on prevention intention. The main effect of message framing was significant, *F*(3,1682) = 4.54, *p* = 0.004, $\eta_{\text{p}}^{2}$ = 0.008, 95% CI [0.001, 0.017] (Figure 4). Multiple comparisons using Holm’s method showed that the mean levels of all three treatment conditions were significantly higher than those of the control condition (*M* = 72.67, *SD* = 16.85), vs. personal condition: *M* = 76.36, *SD* = 18.42, *t*(1682) = 3.13, *p* = 0.011, *d* = 0.209, 95% CI [0.073, 0.345]; vs. public condition: *M* = 75.86, *SD* = 17.95, *t*(1682) = 2.67, *p* = 0.031, *d* = 0.181, 95% CI [0.045, 0.316]; vs. family condition: *M* = 76.31, *SD* = 17.27, *t*(1682) = 3.00, *p* = 0.014, *d* = 0.206, 95% CI [0.071, 0.342]. As in Study 1, no significant difference between the three treatments was obtained, *p*s > 0.971, *d*s < 0.029.

To confirm whether controlling for the social desirability score alters these results, we conducted an ANCOVA including social desirability as a covariate. The results demonstrated significant main effects for the conditions, *F*(3,1681) = 4.68, *p* = 0.003, $\eta_{\text{p}}^{2}$ = 0.008, 95% CI [0.001, 0.018] as well as for social desirability, *F*(1,1681) = 47.58, *p* < 0.001, $\eta_{\text{p}}^{2}$ = 0.028, 95% CI [0.014, 0.045]. Next, multiple comparisons were conducted using Holm’s method. The mean levels of all three treatment conditions were significantly higher than that of the control condition (*M* = 72.67, *SD* = 17.38), vs. personal condition: *M* = 76.46, *SD* = 17.39, *t*(1681) = 3.26, *p* = 0.007, *d* = 0.218, 95% CI [0.082, 0.354]; vs. public condition: *M* = 75.83, *SD* = 17.38, *t*(1681) = 2.69, *p* = 0.029, *d* = 0.182, 95% CI [0.046, 0.317]; vs. family condition: *M* = 76.22, *SD* = 17.38, *t*(1681) = 2.96, *p* = 0.016, *d* = 0.204, 95% CI [0.068, 0.340]. In line with the results of the ANOVA, there were no significant differences between the three treatments, *p*s > 0.842, *d*s < 0.036.

Further, we examined the effect of messaging on each type of perceived threat of the coronavirus by conducting one-way ANOVAs separately. There was a significant main effect on the perceived personal threat, *F*(3,1682) = 3.04, *p* = 0.028, $\eta_{\text{p}}^{2}$ = 0.005, 95% CI [0.000, 0.013] and community threat, *F*(3,1682) = 2.70, *p* = 0.044, $\eta_{\text{p}}^{2}$ = 0.005, 95% CI [0.000, 0.012]; however, no significant main effect on the perceived family threat was found, *F*(3,1682) = 0.18, *p* = 0.910, $\eta_{\text{p}}^{2}$ = 0.000, 95% CI [0.000, 0.002]. Despite the significant main effects of the conditions on both perceived personal and community threats, we could not find a significant difference between conditions with multiple comparisons using Holm’s method, *p*s > 0.052, *d*s < 0.181^6^.

Finally, we found that neither relational mobility nor FNE played a significant moderating role in the relationship between messaging and prevention intention. As in Study 1, two-way ANOVA (4 [condition: personal vs. public vs. family vs. control] × 2 [relational mobility: high vs. low]) was performed. The results showed a significant main effect for conditions, *F*(3,1678) = 4.64, *p* = 0.003, $\eta_{\text{p}}^{2}$ = 0.008, 95% CI [0.001, 0.017], but no significant main effect for dummy-coded relational mobility, *F*(1,1678) = 0.84, *p* = 0.358, $\eta_{\text{p}}^{2}$ = 0.001, 95% CI [0.000, 0.005], nor a significant interaction, *F*(3,1678) = 2.36, *p* = 0.070, $\eta_{\text{p}}^{2}$ = 0.004, 95% CI [0.000, 0.011]. Similarly, the moderating role of FNE on the messaging–prevention relationship was examined using two-way ANOVA (4 [condition: personal vs. public vs. personal + public vs. control] × 2 [FNE: high vs. low]). The results again showed only a significant main effect for the conditions, *F*(3,1678) = 4.57, *p* = 0.003, $\eta_{\text{p}}^{2}$ = 0.008, 95% CI [0.001, 0.017], but no significant main effect for dummy-coded FNE, *F*(1,1678) = 0.02, *p* = 0.889, $\eta_{\text{p}}^{2}$ = 0.000, 95% CI [0.000, 0.002], nor significant interaction, *F*(3,1678) = 1.18, *p* = 0.315, $\eta_{\text{p}}^{2}$ = 0.002, 95% CI [0.000, 0.007]. A series of regression analyses still found no significant interactions of these variables.

### Discussion

In addition to the replication of Study 1, in Study 2, we investigated whether the advantage of prosocially framed messaging could vary when the social responsibility for one’s own close others (i.e., family) became prominent. The results demonstrated that, although exposure to messages promoted intentions more effectively compared with no exposure to messages, the effectiveness of message framing did not vary across treatments. This pattern was confirmed even when social desirability was controlled. Consistent with previous studies, messaging treatments did not have a significant effect on the perceived threats of the coronavirus. Relational mobility and FNE did not moderate the treatment–prevention relationship. These results imply the robustness of the promotional effect of persuasive messaging on the public’s prevention intention, whereas the social context might affect the relative advantage of prosocial appeals.

## General Discussion

The aim of our research was to examine the cultural universality and robustness of the findings of Jordan et al. (2020) and attempt to extend them to the Japanese context. Moreover, we sought to unpack one possible psychological mechanism underlying the impact of the relative advantage of prosocial appeals on prevention intentions against COVID-19 by examining the moderating role of relational mobility and FNE. Although we found a consistent effect of the treatments on prevention intention as Jordan et al. (2020) across the two studies, the relative effectiveness of prosocial over self-interested messaging was not observed. Although our results were inconsistent with those of their earlier set of studies, they were consistent with those of their later set of studies. In addition, emphasizing the benefits of family enhanced prevention intention although relative effectiveness over personal or public treatments was not confirmed. These results were obtained even after controlling for participants’ social desirability, thereby suggesting that the effect of messaging could not be explained by the increase in social pressure to be socially desirable. In sum, the current research partially supports and extends the findings of Jordan et al. (2020) in diverse cultural backgrounds and situations of the spread of infection.

That our results were consistent with only the later studies of Jordan et al. (2020) might be due to the fact that those experiments were conducted in the immediate post-phase of the “early stage of a domestic pandemic.” On March 11, WHO assessed that COVID-19 could be characterized as a pandemic. The American government issued a national emergency declaration on March 13, and several states (e.g., California, New York) subsequently decided to initiate lockdown. The Japanese government declared a state of emergency in seven prefectures on April 7, and the subject area was extended nationwide on April 16. Therefore, we could assume that the data collection for those studies was conducted after the public’s sense of urgency toward COVID-19 had been sufficiently raised. Public awareness of infection control may have already been fixed at a high level during this period. In fact, a national poll demonstrated that 47% of Americans perceived the coronavirus outbreak as a major threat to the health of the entire U.S. population from March 10 to 16; this percentage rose to 66% from March 19 to 24. This rising pattern was also observed in the perceived threat to personal health (i.e., from 27% to 36%; Pew Research Center, 2020). In Japan, 66.6% of Japanese reported that they felt anxiety about COVID-19 from March 6 to 9; this percentage rose to 83.4% from April 3 to 6 and slightly fell to 75.8% from May 29 to June 2 (Survey Research Center, 2020). Under these circumstances, as engaging in infection control itself was regarded as critical by citizens, for *whom* one should act might not have mattered.

However, some reports indicate a different result pattern for the effectiveness of self-interested versus prosocial appeals. Capraro and Barcelo (2020) found that highlighting the “your community” message solely promoted the intention to wear a face-covering compared with the baseline through an online experiment conducted from the end of April to the beginning of May with American citizens. There was no significant difference between the other-oriented messaging (i.e., “your family” and “your country” treatments), self-oriented messaging, and baseline. Further, the relative effectiveness of the “your community” condition over the “your family” condition was verified although the effect was marginally significant. However, the significant effect of all types of messaging was not confirmed when the dependent variable was the intention to practice physical distancing. In contrast to our findings, this study did not find a significant effect of the family condition compared with the baseline. As mentioned before, experiments conducted on Japanese samples from the end of April to early May did not provide consistent results either (Sasaki et al., 2020). We speculate that one possible explanation for these inconsistencies might be the participants’ lack of sufficient attention to the given messages of the experiments (i.e., experimental stimulus). Unlike our studies, whether participants carefully read and comprehended the content of the messages was not checked in their studies. Further examination is essential, using more rigorous procedures to assess the true effect of messaging.

Our hypothesis on the moderating role of relational mobility and FNE was not supported^7^. This result suggests that individual differences in rejection sensitivity were not necessarily associated with prevention intentions after decreasing the risk of interpersonal transmission of COVID-19, which has become widely encouraged in society. From the beginning of the pandemic, a number of scholars have addressed clarifying an effective way to appeal to the public, such as self-focused and other-focused framed messages, norm-based messages (Bilancini et al., 2020), and messages that appeal to one’s reasoning and emotion (Capraro and Barcelo, in press). As engaging in COVID-19 prevention behavior contributes to preventing the spread of infection, it protects not only oneself, but also indirectly others. Therefore, prevention behavior can be regarded as prosocial behavior. Given that empathy is associated with prosocial behavior and cooperation (e.g., Eisenberg and Miller, 1987), it may indeed lead to prevention behaviors as already suggested by some researchers (Christner et al., in press; Lunn et al., 2020; Pfattheicher et al., 2020). Sakakibara and Ozono (2020) provided suggestive findings with respect to the psychological factors that promote prevention behaviors via a survey conducted from the end of April to the beginning of May with a Japanese sample^8^. Although the motivation to protect oneself and others from infection had a significant positive effect on prevention behaviors, perceived social norms and the motivation to avoid negative evaluation by others did not have a significant impact. Furthermore, of the two aspects of interdependent self-construal (Hashimoto and Yamagishi, 2013, 2016), *harmony seeking* (i.e., willingness to seek harmonious relationships with others) was positively linked to prevention behaviors, and *rejection avoidance* (i.e., willingness to avoid being disliked and not accepted by others) was not. These results strongly endorse our notion that for whom one should act became less important after prevention behavior became common among people and that the public’s prevention behaviors may be guided more strongly by the motivation to cooperate with others rather than concerns for social rejection.

Our findings may provide implications for the field regarding the impact of culture on persuasion. Specifically, the results show a contradictory pattern with those of prior studies. Drawing upon the literature on personalized matching, our studies provide inconsistent findings. This might reflect the fact that the advantage of personalized matching might disappear after people are chronically exposed to the contagious threat and have a better understanding of the issue. We should note, however, that health behaviors that have been addressed in previous studies were focused on those whose consequences have an impact only within individuals and do not ripple to other individuals, such as flossing (Uskul et al., 2009), caffeine consumption (Uskul and Oyserman, 2010), and cervical cancer screening (Spina et al., 2018). As COVID-19 transmits from human to human, an individual’s prevention behavior is key to breaking the chain of transmission. Therefore, COVID-19 prevention behaviors assume the character of making people aware of social relationships. As this may determine the effectiveness of messaging, further verification is required.

Our findings serve as a practical contribution for infection control of COVID-19. Personal prevention behaviors (e.g., avoiding the three Cs: closed spaces, crowded places, and close-contact settings) appeared to be prevalent from mid to late May in Japan. Considering the associative network model (Hastie and Kumar, 1979), we could interpret that messaging may have activated knowledge on infection control, resulting in the promotion of prevention intention. As indicated by the Survey Research Center (2020), citizens’ sense of anxiety or urgency may fade with time, leading to less prevention behavior. Therefore, governors should remember to remind the public of the threat of COVID-19 from time to time to avoid the public becoming less alert.

We note the limitations of the current research. First, although we demonstrate the significant influence of messaging on prevention intention, this intention is not necessarily consistent with actual prevention behavior. However, Gollwitzer et al. (2020) show that self-reported social distancing was linked to actual health behavior during the early stage of the first wave of the COVID-19 pandemic in the United States. This suggests that our findings can be a valid indication of the effectiveness of messaging on actual prevention behaviors. Second, the participants in our studies (i.e., CrowdWorks samples) might represent a specific population of Internet users in Japan. Future research using a field experiment or a natural experiment so that the effectiveness of messaging can be examined with a more representative sample is recommended.

## Conclusion

Our studies demonstrate that persuasive messages encourage prevention intentions even after some degree of public awareness of COVID-19 infection prevention has been sufficiently heightened. After acquiring basic knowledge of the COVID-19 pandemic, and once infection control is widely ingrained in society, the continuous dissemination of such information might be evaluated as less important. Considering the current findings, however, prevention messaging can still have a significant impact on prevention intention even after the importance of infection control by individuals has become a widespread social concern. The fight against COVID-19 shall be long; thus, intermittent prevention messaging, regardless of self-interested or prosocial framing, would contribute to keeping the public on its guard against the disease, and promote preventive behaviors.

## Data Availability Statement

The datasets presented in this study can be found in online repositories. The names of the repository/repositories and accession number(s) can be found below: https://osf.io/m2hu9.

## Ethics Statement

The studies involving human participants were reviewed and approved by The Ethical Committee of Nara University. Written informed consent for participation was not required for this study in accordance with the national legislation and the institutional requirements.

## Author Contributions

TM and FM designed the study and administered the experiments. TM conducted the data analysis. TM and FM wrote and revised the manuscript. Both authors contributed to the article and approved the submitted version.

## Conflict of Interest

The authors declare that the research was conducted in the absence of any commercial or financial relationships that could be construed as a potential conflict of interest.

## Footnotes

1 https://osf.io/m2hu9

2 https://crowdworks.jp

3 The pictogram used in the slides was developed by the Hokkaido government to encourage citizens’ infection control (http://www.pref.hokkaido.lg.jp/ss/tkk/0514pictogram.htm).

4 As dichotomizing continuous variables may reduce statistical power, we also ran a series of regression analyses with each comparison to test the interactions. Here, we still found no significant interactions.

5 One participant reported participating in the experiment twice because of the failure to send response data by a machine error in the first experiment. As this participant was assigned to two different conditions, the data were eliminated from the subsequent analysis.

6 Defining perceived personal threat as a dependent variable, the mean difference between the family condition (*M* = 74.07, *SD* = 23.69) and control condition (*M* = 69.70, *SD* = 24.03) was marginally significant, *t*(1682) = 2.63, *p* = 0.052, *d* = 0.181, 95% CI [0.046, 0.317]. In addition, including perceived community threat as a dependent variable, the mean difference between family condition (*M* = 78.37, *SD* = 22.21) and control condition (*M* = 74.39, *SD* = 22.35) was marginally significant, *t*(1682) = 2.58, *p* = 0.061, *d* = 0.177, 95% CI [0.042, 0.313]. None of the other differences between the conditions were even marginally significant.

7 We should note that the fact that relational mobility within-country did not play a moderating role does not immediately mean that it would not play a role across cultures as well. Thus, comparative research that examines the moderating role across cultures is required.

8 In this study, the authors set a few items to detect satisficers and only participants who could clear these items were included in the analysis.
